# CoenzymeQ10 and Ischemic Preconditioning Potentially Prevent Tourniquet-Induced Ischemia/Reperfusion in Knee Arthroplasty, but Combined Pretreatment Possibly Neutralizes Their Beneficial Effects

**DOI:** 10.3390/antiox11020419

**Published:** 2022-02-18

**Authors:** Prangmalee Leurcharusmee, Passakorn Sawaddiruk, Yodying Punjasawadwong, Nantawit Sugundhavesa, Kasisin Klunklin, Siam Tongprasert, Patraporn Sitilertpisan, Thidarat Jaiwongkam, Nattayaporn Apaijai, Nipon Chattipakorn, Siriporn C. Chattipakorn

**Affiliations:** 1 Neurophysiology Unit, Cardiac Electrophysiology Research and Training Center, Faculty of Medicine, Chiang Mai University, Chiang Mai 50200, Thailand; prangmalee.l@cmu.ac.th (P.L.); passakorn.s@cmu.ac.th (P.S.); thidarat.j@cmu.ac.th (T.J.); nattayaporn.a@cmu.ac.th (N.A.); nipon.chat@cmu.ac.th (N.C.); 2 Center of Excellence in Cardiac Electrophysiology Research, Chiang Mai University, Chiang Mai 50200, Thailand; 3 Department of Anesthesiology, Faculty of Medicine, Chiang Mai University, Chiang Mai 50200, Thailand; yodying.punjasa@cmu.ac.th; 4 Department of Orthopedics, Faculty of Medicine, Chiang Mai University, Chiang Mai 50200, Thailand; nantawit.s@cmu.ac.th (N.S.); kasisin.k@cmu.ac.th (K.K.); 5 Department of Rehabilitation, Faculty of Medicine, Chiang Mai University, Chiang Mai 50200, Thailand; siam.tongpr@cmu.ac.th; 6 Faculty of Associated Medical Science, Chiang Mai University, Chiang Mai 50200, Thailand; patraporn.s@cmu.ac.th; 7 Cardiac Electrophysiology Unit, Department of Physiology, Faculty of Medicine, Chiang Mai University, Chiang Mai 50200, Thailand; 8 Department of Oral Biology and Diagnostic Sciences, Faculty of Dentistry, Chiang Mai University, Chiang Mai 50200, Thailand

**Keywords:** antioxidants, arthroplasty, ischemic preconditioning, mitochondria, pain, tourniquet

## Abstract

Tourniquet (TQ) use during total knee arthroplasty (TKA) induces ischemia/reperfusion (I/R) injury, resulting in mitochondrial dysfunction. This study aims to determine the effects of coenzyme Q10 (CoQ10) and ischemic preconditioning (IPC), either alone or in combination, on I/R-induced mitochondrial respiration alteration in peripheral blood mononuclear cells (PBMCs) and pain following TKA. Forty-four patients were allocated into four groups: control, CoQ10, IPC, and CoQ10 + IPC. CoQ10 dose was 300 mg/day for 28 days. IPC protocol was three cycles of 5/5-min I/R time. Mitochondrial oxygen consumption rates (OCRs) of PBMCs were measured seven times, at baseline and during ischemic/reperfusion phases, with XFe 96 extracellular flux analyzer. Postoperative pain was assessed for 48 h. CoQ10 improved baseline mitochondrial uncoupling state; however, changes in OCRs during the early phase of I/R were not significantly different from the placebo. Compared to ischemic data, IPC transiently increased basal OCR and ATP production at 2 h after reperfusion. Clinically, CoQ10 significantly decreased pain scores and morphine requirements at 24 h. CoQ10 + IPC abolished analgesic effect of CoQ10 and mitochondrial protection of IPC. In TKA with TQ, IPC enhanced mitochondrial function by a transient increase in basal and ATP-linked respiration, and CoQ10 provides postoperative analgesic effect. Surprisingly, CoQ10 + IPC interferes with beneficial effects of each intervention.

## 1. Introduction

Total knee arthroplasty (TKA), an effective surgical treatment for end-stage knee osteoarthritis (OA), successfully provides pain relief and improves the quality of life [1]. Perioperative care of TKA becomes more challenging in patients with increasing age and multiple comorbidities [1,2]. Also, TKA has a trend toward an ambulatory setting [3,4]. Thus, besides a current anesthesia practice, alternative strategies to enhance function at the cellular levels are essential as an adjunct therapy to improve postoperative clinical outcomes. 

Tourniquet (TQ) application in TKA has been a controversial issue [5]. Previous studies have associated intraoperative TQ use with a reduction in intraoperative blood loss and postoperative blood transfusion [6,7]. However, other studies reported that TQ is related to several disadvantages, including early postoperative pain [8], delayed rehabilitation [9], and ischemia and reperfusion (I/R) injury to the insulted skeletal muscle as well as remote vital organs [10]. Although, the mechanisms underlying the TQ-induced skeletal muscle I/R injury are complex and not thoroughly understood, dysfunction of the mitochondria is considered the main source [10,11]. 

Mitochondria are multifunctional organelles that play major roles in fundamental cellular processes including cellular bioenergetic, redox status, calcium homeostasis, cell survival, and apoptosis. External stressors, such as the I/R process, impair physiologic regulation of mitochondrial bioenergetics, resulting in mitochondrial dysfunction [11]. The effects of skeletal muscle I/R on cellular respiration have been scantly investigated. In a murine model of acute hindlimb I/R, the activities of mitochondrial electron transport chain (ETC) complexes of the gastrocnemius muscle were decreased after 3-h ischemia followed by 4-h reperfusion [12]. In addition to changes in the skeletal muscle itself, the maximal respiration of mitochondria isolated from lung parenchyma decreased after mice were subjected to 2-h ischemia of both hindlimbs, followed by 2-h reperfusion [13]. In TKA patients, one previous study has shown a preserved amount and function of the mitochondrial ETC complexes I-III of the vastus medialis muscle at a 60-min ischemic time [14]. To date, mitochondrial bioenergetics of the peripheral blood mononuclear cells (PBMCs) during ischemic and reperfusion periods in TKA patients with TQ application remain to be elucidated.

Several pharmacological and interventional strategies to prevent or attenuate the TQ-related I/R injury in TKA have been investigated and a comprehensive review reported that antioxidants and ischemic preconditioning potentially exerted positive effects [10]. 

Coenzyme Q10 (CoQ10) is an electron carrier in the mitochondrial ETC, transferring electrons from complex I and II to complex III during oxidative phosphorylation (OXPHOS). It also prevents cellular damage by exerting potent antioxidative activity [15]. Depletion of endogenous CoQ10 content in mitochondria of the skeletal muscle occurs in aging [16]. Fortunately, exogenous CoQ10 supplementation restores plasma CoQ10 concentration [17], enhances mitochondrial respiration [18], and demonstrates protective effects against myocardial I/R injury [19], as well as damage during cardiopulmonary bypass [20]. 

Ischemic preconditioning (IPC) is an intervention where the application of one or more brief episodes of ischemia and reperfusion generates small amounts of free radicals and subsequently increases tissue toleration to prolonged ischemic stress and reperfusion injury. There are two phases of IPC protection. Acute preconditioning occurs within minutes and disappears within hours, while the delayed phase of protection initiates after 24 h and persists for several days [21,22]. The acute phase of IPC activates cytoprotective processes, such as antiapoptotic, prosurvival MAPK/ERK kinase and PI3K/AKT/PKB pathways, inhibits mitochondrial permeability transition pores (mPTPs) opening, and exerts cardioprotection in myocardial I/R [23,24]. In TKA patients, IPC induces protective genomic responses [25] and potentially improves postoperative pain control [26,27]. 

Thus, the objectives of this study were (1) to determine the effects of preoperative CoQ10 supplementation on mitochondrial respiration in chronic knee OA patients; (2) to examine the efficacy of the two independent interventions, which were CoQ10 and IPC, during the early phase of TQ-induced I/R; (3) to detect possible interactions between these two factors; and (4) to demonstrate the effects of postoperative CoQ10 on the late phase of TQ-induced I/R. We hypothesized that (1) preoperative CoQ10 improved an impaired mitochondrial function of OA patients at the baseline, and postoperative CoQ10 restored mitochondrial dysfunction related to TQ-induced I/R; and (2) exogenous CoQ10 and IPC, either alone or in combination, were more effective than placebo in preserving mitochondrial oxygen consumption rates (OCRs) and alleviating pain following TKA. 

## 2. Materials and Methods

### 2.1. Study Design

This is a randomized, triple-blinded, placebo-controlled trial with a 2 × 2 factorial design. This study was registered at clinicaltrials.in.th (TCTR20190128001) on 24 January 2019 and approved by the Ethics Committee of Faculty of Medicine, Chiang Mai University on 30 January 2019 (Ethical-Approval number: ANE-2564-05663). This manuscript adhered to the Consolidated Standards of Reporting Trials (CONSORT) statement. 

### 2.2. Participants

After providing a written informed consent, 60- to 80-year-old patients with primary knee OA scheduled for elective unilateral TKA were included in the trial. The exclusion criteria were body mass index > 35 kg/m^2^, any possible high oxidative stress conditions including malignancy or infectious diseases, neurodegenerative diseases, atherosclerotic diseases, chronic kidney disease, active heavy smoking, recent use of antioxidant (within four weeks) or glucocorticoids (within three months), neurocognitive disorders, and allergy to any medications used in the study protocol. Forty-four patients were recruited from 31 January 2019 to the end of August 2020. 

### 2.3. Study Interventions, Randomization and Blinding

The first intervention was CoQ10. Exogenous CoQ10 was administered for one month throughout the perioperative period. To determine the effects of preoperative and postoperative CoQ10 supplements, biochemical outcomes were assessed at two weeks before surgery, on the day of surgery, and at two weeks after surgery. 

The second intervention was IPC. The IPC was randomly assigned to all patients regardless of CoQ10 supplementation. Consequently, there were four study groups which were control (placebo + sham IPC), CoQ10 alone (CoQ10 + sham IPC), IPC alone (placebo + IPC), and a combined intervention (CoQ10 + IPC). To determine the effects of classical or acute phase of IPC protection, biochemical and clinical outcomes were assessed within the first few hours after IPC application. 

One month before surgery, a research coordinator screened patients at a pre-anesthesia clinic. After they consented to the study, the patients were randomly allocated to receive either CoQ10 or placebo. The computer-generated random numbers with a block size of four were contained in sealed opaque envelopes. A research coordinator opened the envelope and prepared an unlabeled bottle of study drugs. Both CoQ10 and the identical placebo soft gel were manufactured by MEGA Lifesciences Public Company Limited, Samutprakarn, Thailand. The patients in the CoQ10 group were assigned to ingest a 100-mg CoQ10 soft gel three times per day (300 mg/day) for 14 days before and 14 days after the operation, while those in the placebo group received placebo soft gels with a uniform prescription. 

At the anesthesia induction room one hour before surgery, a research investigator randomized patients to receive either IPC or sham IPC. The computer-generated random numbers with a block size of four were contained in sealed opaque envelopes. A research investigator opened the envelope and performed the IPC or sham IPC. The TQ was applied on the operated thigh of all patients. Patients assigned for the IPC received the same IPC protocol, which was three cycles of 5/5-min I/R time using a pressure of systolic blood pressure (SBP) +100 mmHg, while those assigned for the sham IPC obtained unpressurized TQ for 30 min. 

Preoperative, intraoperative, and postoperative data and blood samples were collected by two blinded data collectors. Patients, anesthesiologists, nurse anesthetists, surgeons, ward nurses, laboratory physicians, and data analysts were blinded to the group allocation during the entire study. 

### 2.4. Anesthesia Protocol and Operative Procedure

All patients received spinal anesthesia using 0.5% isobaric bupivacaine 10–15 mg. Four to 6 mL/kg of Lactate Ringer’s solution was loaded to prevent hypotension following spinal anesthesia. In addition, femoral triangle block with retained catheter and single-shot IPACK block were performed using 20 mL of 0.25% bupivacaine with 5 µg/mL epinephrine for each injection. Preoperatively, antibiotics and tranexamic acid were administered. Intraoperatively, vital signs were continuously monitored and maintained at the patient’s baseline level. Intravenous fluid was provided as a maintenance dose or as needed if there was a significant blood loss. Oxygen was supplemented only if pulse oximetry was less than 94%. If patients required intraoperative sedation, midazolam was administered using the titration technique. Propofol, corticosteroids and local periarticular infiltration were not allowed throughout the perioperative period.

The operation was performed by the two orthopedic surgeons (NS and KK) using a standard medial parapatellar approach and cemented TKA. The thigh TQ was inflated at SBP + 100 mmHg before an anterior longitudinal midline incision, and it was released after the closure of parapatellar arthrotomy. 

### 2.5. Postoperative Analgesic Protocol

All patients received standard postoperative pain medications including 1 gm of oral acetaminophen every 6 h and 90 mg of Arcoxia every 24 h for 48 h. Ten mL of 0.25% bupivacaine with 5 µg/mL epinephrine was injected through the femoral triangle catheter every 12 h for 24 h. Intravenous morphine (2–4 mg/dose depending on patient’s weight) was administered when patients experienced moderate to severe pain. 

### 2.6. Biochemical Outcomes

**Figure 1:**
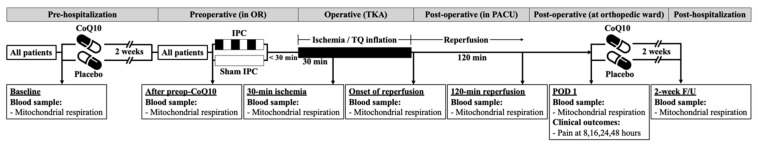
Study flow demonstrating patient allocation, study protocols, and outcome measurements.

The primary outcomes were mitochondrial OCRs of PBMCs. Venous blood samples were collected seven times (Figure 1). The baseline specimen was obtained two weeks before surgery (T0: baseline) at the preanesthetic clinic. Thereafter, to assess the effect of preoperative CoQ10 supplementation, venous blood was drawn on the day of surgery before the IPC or sham IPC was performed (T1: pre-operation). To compare the effects of the two interventions on the early phase of TQ-induced I/R, blood samples were collected at 30 min after TQ inflation (T2: 30-min ischemia), at the onset of TQ release (T3: onset of reperfusion), and at two hours after reperfusion (T4: 2-h reperfusion). Lastly, to measure the effect of CoQ10 on the late phase of reperfusion, specimens were collected at 24 h (T5: 24-h reperfusion) and two weeks after surgery (T6: 2-week post-operation).

The mitochondrial OCR, which is an indicator of OXPHOS, was measured in the PBMCs. Once plasma was collected, PBMCs were isolated using a Ficoll density gradient centrifugation technique. The measurement of OCR was carried out by using the mitochondrial stress test assay (Seahorse XF Cell Mito Stress Test Kit) and the Seahorse XFe 96 Extracellular Flux Analyzer (Agilent Technologies, Santa Clara, CA, USA) according to the manufacturer’s protocol. Briefly, the PBMCs were loaded into the XFe96-well plate and the OCR was measured under basal conditions (basal respiration) and in response to subsequently added reagents. One µM-Oligomycin, 2 µM-FCCP, and 0.5 µM-Rotenone/antimycin A were sequentially added to measure proton leak, maximal, and non-mitochondrial respiration rates, respectively. The basal respiration rate is a baseline oxygen consumption used by the ETC complex IV to establish a proton gradient across the inner mitochondrial membrane. To maintain the proton current balance under physiologic conditions, protons re-entry to the mitochondrial matrix dominantly by the adenosine triphosphate (ATP)-generating process through the ATP synthase (complex V) and partly by the uncoupling protein-mediated proton leaks [28]. Accordingly, after administration of 1 µM-Oligomicin, which is an ATP synthase inhibitor, the ATP-related respiration was suppressed, and the basal proton leak respiration rate was measured. The ATP production (ATP-linked respiration) was calculated by subtracting proton leak respiration from basal respiration. Additionally, the coupling efficiency was calculated by dividing the ATP-linked respiration by basal respiration. 

### 2.7. Clinical Outcomes

The secondary outcomes were postoperative pain scores and morphine consumption. Postoperative pain was assessed using a numeric rating scale (NRS) by the ward nurses every four hours. When a patient experienced moderate to severe pain (NRS ≥ 4), ward nurses administered intravenous morphine and the cumulative morphine requirement was evaluated at 8, 16, 24, and 48 h after surgery. 

### 2.8. Statistical Analysis

Based on our preliminary measurements of ATP-related respiration rate at 30-min ischemia (69.2 ± 19.6 pmol/min) and 2-h reperfusion (77.2 ± 23.2 pmol/min) in TKA patients receiving no study intervention, we estimated the sample size for a one-sample mean test. Ten patients per group were required to identify a 30% difference between groups, with 5% type-1 error and 80% power. 

Data were analyzed using STATA 16 software (StataCorp LLC, College Station, TX, USA). Continuous variables were examined for normality using the Shapiro–Wilk normality test. Unpaired t-test or Wilcoxon rank-sum test was performed to assess differences between two groups, while one-way ANOVA with Bonferroni multiple comparison test or Kruskal–Wallis rank test was used to assess differences among four study groups. Linear regression with interaction was used to analyze the interaction terms of continuous parameters between the CoQ10 and IPC. Categorical data were assessed for differences using Fisher’s exact probability test. Differences associated with *p*-value < 0.05 were considered significant.

## 3. Results

**Figure 2:**
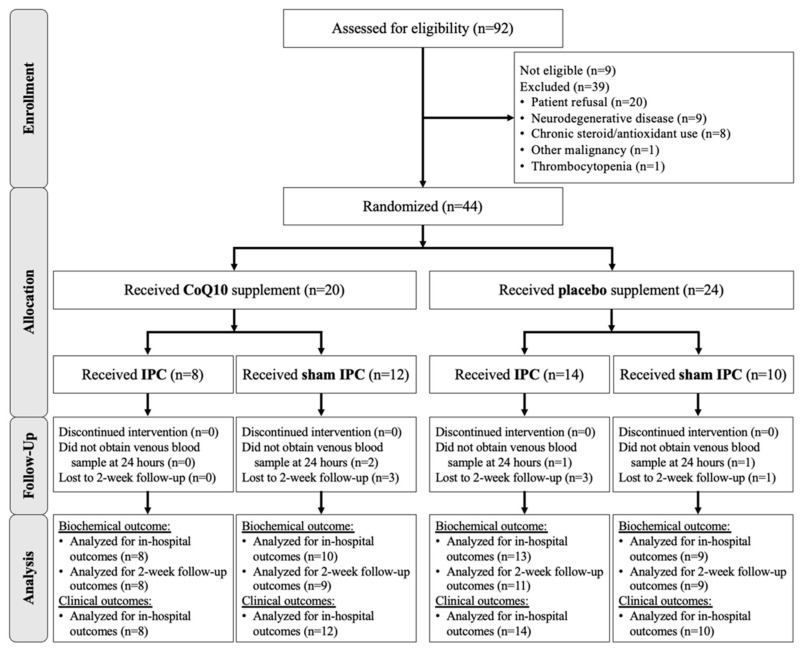
CONSORT flow diagram for screening, inclusion, exclusion, and lost to follow-up of the study participants.

A total of 92 patients were screened for inclusion. Of these, nine patients were not eligible, and 39 patients were excluded, leaving 44 patients for randomization (Figure 2). Since the first randomization, all patients received the medication and intervention as allocated and all data were analyzed as intended to treat. Demographic and intraoperative data of patients receiving placebo or CoQ10 were similar, except for the age range where those receiving CoQ10 were significantly older by chance (Table 1). 

### 3.1. Effects of Preoperative CoQ10 Supplementation on Baseline Conditions of Knee OA Patients

**Figure 3:**
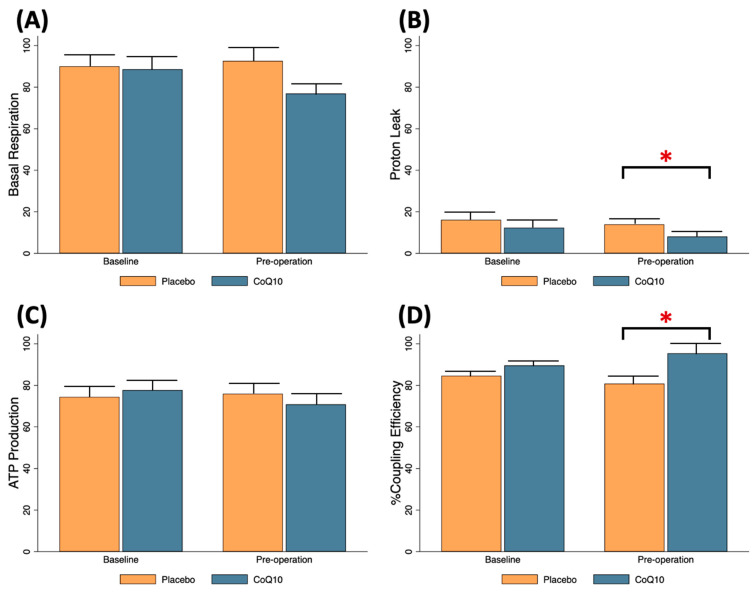
Mitochondrial respiration alteration following 2-week preoperative CoQ10 supplementation. (**A**) basal respiration, (**B**) proton leak, (**C**) ATP production, (**D**) % coupling efficiency. * *p* < 0.05 between CoQ10 and placebo groups.

Baseline mitochondrial respiration parameters including basal respiration, proton leak, ATP production, and % coupling efficiency were comparable between the patients receiving placebo and those obtaining CoQ10 (Figure 3). After two weeks of CoQ10 ingestion, proton leak decreased (Mean ± SE; placebo 14.0 ± 2.0 vs. CoQ10 8.1 ± 1.4 pmol/min, *p* = 0.028) and % coupling efficiency increased (Mean ± SE; placebo 80.8 ± 3.8 vs. CoQ10 95.4 ± 5.4 pmol/min, *p* = 0.012) when compared to the placebo group, indicating that exogenous CoQ10 improved ADP-driven respiration and uncoupling of OXPHOS, which occurred at baseline condition of knee OA patients (Figure 3B,D). 

### 3.2. Effects of CoQ10, IPC, and CoQ10 + IPC on Early Phase of TQ-Induced I/R

**Figure 4:**
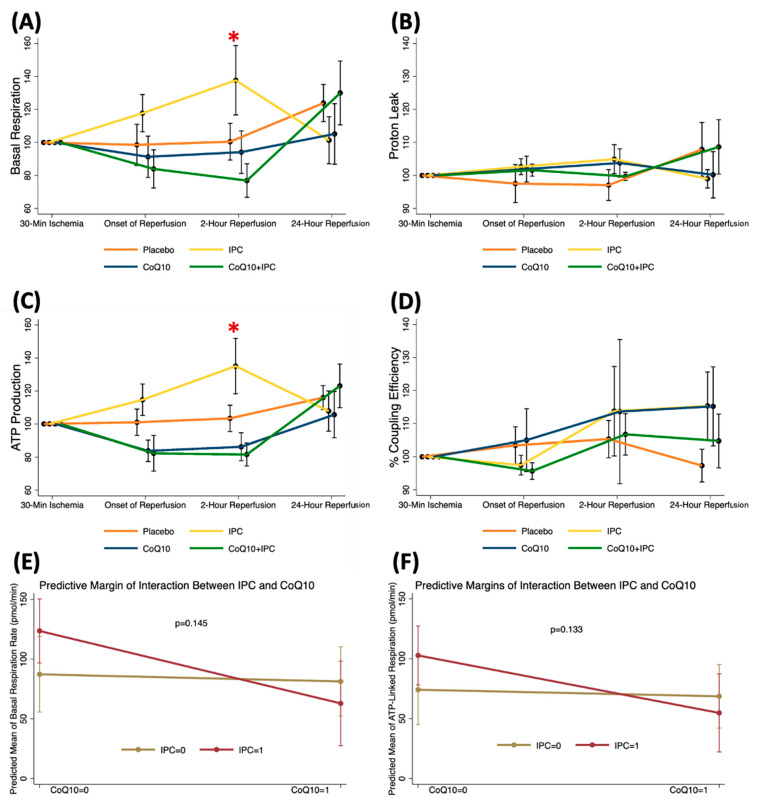
Mitochondrial respiration alteration following early phase of tourniquet-induced ischemia and reperfusion in TKA patients receiving placebo vs. IPC vs. CoQ10 vs. CoQ10 + IPC. (**A**) basal respiration, (**B**) proton leak, (**C**) ATP production, (**D**) % coupling efficiency, (**E**) predicted mean of basal respiration, (**F**) predicted mean of ATP production. Results are presented as differences from ischemic data. * *p* < 0.05 between 2-h reperfusion and 30-min ischemic data in the IPC group.

At the 30-min ischemic time, mitochondrial respiration parameters were comparable among the patients receiving no intervention (control), CoQ10, IPC, and CoQ10 + IPC. When compared to the ischemic data, there were no significant changes in mitochondrial respiration throughout the reperfusion periods in patients receiving CoQ10 and the combined intervention (Figure 4). Interestingly, the IPC improved the mitochondrial basal respiration (Mean ± SE; 30-min ischemia 82.9 ± 10.0 vs. 2-h after reperfusion 120.6 ± 21.7 pmol/min, *p* = 0.036) and ATP production (Mean ± SE; 30-min ischemia 67.8 ± 8.5 vs. 2-h after reperfusion 102.7 ± 18.5 pmol/min, *p* = 0.016) at 2 h after reperfusion (Figure 4A,C). These findings indicated that the IPC showed transient protective effects on mitochondrial function. Among patients receiving the IPC, the beneficial effects on the mitochondrial OCRs were demonstrated only when CoQ10 was not pretreated. We speculated that CoQ10 might interfere with the protective mechanisms of the IPC. However, the interaction between the IPC and CoQ10 on the basal respiration and ATP production at 2 h after reperfusion was not significant (Figure 4E,F). 

### 3.3. Effects of CoQ10, IPC, and CoQ10 + IPC on Postoperative Analgesia

**Figure 5:**
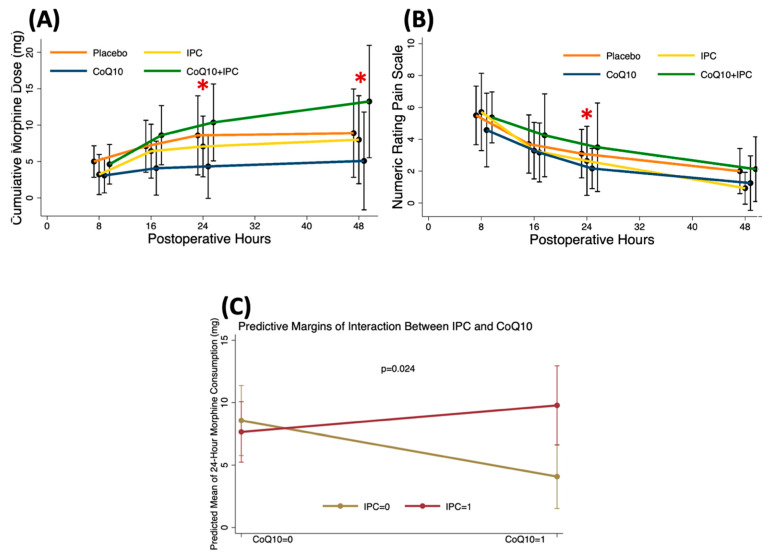
Postoperative pain during the first 48 h in TKA patients receiving placebo vs. IPC vs. CoQ10 vs. CoQ10 + IPC. (**A**) morphine consumption, (**B**) pain score, (**C**) predicted mean of 24 h morphine consumption. * *p* < 0.05 among the four groups.

Clinically, postoperative morphine consumption at 24 and 48 h was significantly lower in the CoQ10 group when compared to the control and CoQ10 + IPC groups (Median [P25-P75] at 24 h; CoQ10 3.5 [1,2,3,4,5,6] vs. placebo 6 [5,6,7,8,9,10,11,12,13,14,15] vs. CoQ10 + IPC 9 [6–13.5] mg, *p* = 0.037) (Figure 5A). In accordance with the cumulative morphine dose, the NRS at 24 h was significantly lower in the CoQ10 group when compared to the placebo and the CoQ10 + IPC groups (Median [P25-P75]; CoQ10 2.5 [2,3,4] vs. placebo 5.5 [3,4,5,6] vs. CoQ10 + IPC 5.5 [3,4,5,6] mg, *p* = 0.013) (Figure 5B). These results indicated that CoQ10 provided preventive analgesia within the first 48 h after TKA. When compared with the control group, the effects of CoQ10, IPC, or CoQ10 + IPC on 24-h morphine requirements were −4.5 mg (95%CI; −8.4, −0.6 mg, *p* = 0.026), −0.9 mg (95%CI; −4.7, 2.9 mg, *p* = 0.631), and 6.6 mg (95%CI; 0.9, 12.3 mg, *p* = 0.024), respectively (Figure 5C). This finding suggested that IPC counteracted the analgesic effects of the CoQ10. 

### 3.4. Effects of Perioperative CoQ10 Supplementation on Late Phase of TQ-Induced I/R

**Figure 6:**
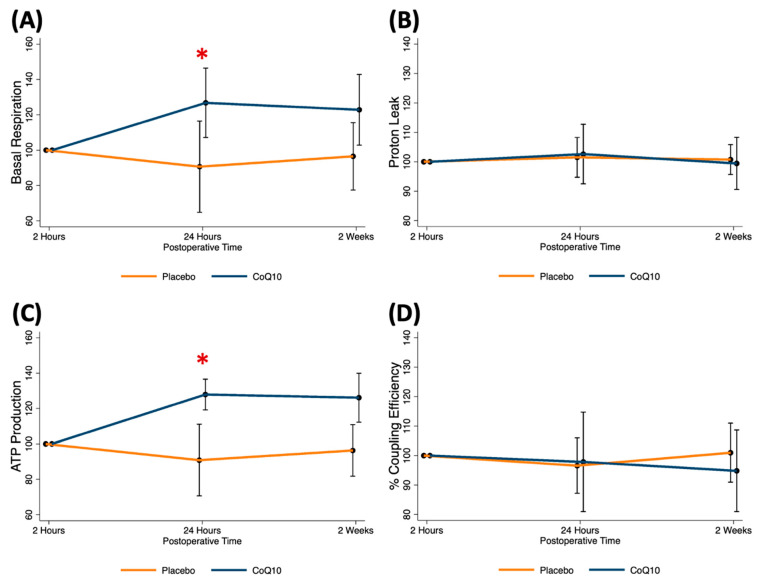
Mitochondrial respiration alteration following late phase of tourniquet-induced ischemia and reperfusion in patients receiving perioperative CoQ10 supplementation. (**A**) basal respiration, (**B**) proton leak, (**C**) ATP production, (**D**) % coupling efficiency. Results are presented as differences from 2-h postoperative data. * *p* < 0.05 between CoQ10 and placebo groups.

When compared to placebo, exogenous CoQ10 restored the mitochondrial basal respiration (Mean difference (95%CI); 48.6 (0.4–96.9) pmol/min, *p* = 0.048) and ATP production (Mean difference (95%CI); 45.7 (11.9–79.5) pmol/min, *p* = 0.008) at 24 h after reperfusion (Figure 6A,C). Ultimately, all mitochondrial parameters were comparable between the patients receiving placebo and those obtaining CoQ10 at two weeks after surgery (Figure 6). 

## 4. Discussion

The present study investigated the effects of CoQ10, IPC, and a combined intervention on TQ-induced I/R during TKA by measuring mitochondrial respiration of PBMCs at the ischemia and various reperfusion periods and assessing postoperative clinical outcomes. Regarding the CoQ10, preoperative 2-week supplement improved baseline mitochondrial uncoupling state of knee OA patients; however, changes in OCRs during the early phase of TQ-induced I/R were not significantly different from the control group. Postoperatively, CoQ10 decreased pain scores and morphine requirements at 24 h. Additionally, the basal and ATP-related OCRs, which were moderately reduced during 2-h reperfusion, were restored at 24 h after surgery. Concerning the IPC, it transiently enhanced mitochondrial basal respiration and ATP production at two hours after reperfusion; however, it showed no clinical benefit. When CoQ10 and IPC were combined, the postoperative analgesic effect of CoQ10 and the protective effects of IPC on mitochondrial function were abolished. These findings suggested a possible interaction between the CoQ10 and IPC. 

Alteration of mitochondrial function conceivably occurs during the I/R in the skeletal muscle [11,29]. Briefly, during TQ inflation or ischemic phase, cessation of blood and oxygen supply reduces aerobic glycolysis and mitochondrial OXPHOS. Attenuation of activities of ETC complexes I, II, and IV during prolonged ischemia leads to a reduction of ATP synthesis, increase in reactive oxygen species (ROS) production, decrease in antioxidant defense, accumulation of cytosolic H^+^ and Ca^2+^, and formation of the mPTPs. During reperfusion, ATP production and mitochondrial membrane potential are restored if the function of the mitochondrial respiratory chain is preserved. However, reoxygenation to the ischemia-insulted myocytes may produce additional cell damage. Fortunately, the deleterious apoptotic events after TQ deflation rarely occur because skeletal muscle is tolerant to ischemia and the I/R injury, which is predominantly determined by the ischemic time [30]. The present study demonstrated that mitochondrial OCRs in PBMCs at 30 min ischemia and during reperfusion periods in the control group were not significantly altered from the preoperative parameters. These results are in accordance with the study of Jawhar A. et al., which reported unchanged activities of the ETC complexes I-III at 60-min TQ time [14]. Besides, a previous in vitro human skeletal muscle model showed that 3-h ischemia followed by 2-h reperfusion resulted in a 50% decrease in cell viability [31]. Therefore, the preserved mitochondrial respiration in the present study possibly resulted from a restricted TQ inflation time which is generally limited to less than two hours in a clinical setting. 

CoQ10 is a potent antioxidant frequently used in clinical practice. It potentially prevents the TQ-induced I/R injury because the CoQ, as part of mitochondrial ETC, regulates ATP and ROS synthesis [12,19,32,33,34]. In aging and chronic arthritis patients, endogenous CoQ10 levels have been shown to significantly decrease, thus inducing mitochondrial vulnerability to I/R processes [19,35]. Exogenous CoQ10 supplementation effectively reduced mitochondrial oxidative stress and possibly restored the ETC activity [36]. In the present study, preoperative CoQ10 ingestion (300 mg/day for 14 days) improved mitochondrial bioenergetics in PBMCs by reducing proton leak and enhancing mitochondrial coupling efficiency. However, preoperative improvement of mitochondrial function in PBMCs did not show significant protection against an early phase of TQ-induced I/R in TKA patients. During a late phase of reperfusion, a restoration of basal and ATP-related mitochondrial OCRs in PBMCs occurred at 24 h after surgery in patients pretreated with CoQ10. An insufficient benefit of the CoQ10 pretreatment during the initial reperfusion periods might result from a small availability of the CoQ10 molecule at the inner mitochondrial membrane, where the ETC is located. In mitochondria, CoQ10 is likely to be localized in the outer membrane because of its strong lipophilic nature [37]. Moreover, plasma CoQ10 concentration following oral CoQ10 supplement relies strongly on the formulation, dosage, and duration of CoQ10 [17,38]. The protective effect of CoQ10 on skeletal muscle I/R might be different with its various preparations. 

Concerning the clinical outcomes, the analgesic effect of CoQ10 in a surgical setting has not been particularly elucidated. In the present study, CoQ10 demonstrated better pain control at 24 h after surgery. However, there was a significant negative interaction effect when the IPC was combined. An anti-nociceptive effect of the CoQ10 has been studied in a rat model of knee OA and the mechanism of pain suppression was suggested to be caused by an anti-inflammation [39]. Previous in vivo study has also shown that CoQ10 pretreatment significantly inhibited TNF-α and NF-κβ activation during I/R of skeletal muscle [33]. 

IPC performed directly on the vessels to be occluded is termed a local IPC. It provides early and late phases of protection [23]. The early phase, as applied in this study, has shown its cytoprotection against TQ-induced I/R injury by improving mitochondrial maximal oxidative capacities and ETC complexes I and II activities, enhancing antioxidant defense, inhibiting mPTP opening related cell death, and reducing expression of genes and proteins involved in apoptosis [25,40,41,42,43]. Consistently, in the present study, IPC demonstrated an increased basal mitochondrial OCR, which was predominantly driven by H^+^ flow through the ATP synthase, at 2 h after TQ deflation. This favorable effect was transient and did not last to 24 h after reperfusion. Referring to the clinical endpoints of knee surgery, preoperative IPC (1 episode of a 5-min TQ inflation followed by a 5 to 10-min TQ release) on the operative thigh demonstrated postoperative analgesic effects [26,27,43]. However, this present study failed to validate this benefit of the IPC. The discrepancy might stem from different IPC protocols since the mechanistic effect of the IPC depends on the number of cycles and duration of ischemia and reperfusion time [44,45]. 

The negative interaction between CoQ10 and IPC on mitochondrial respiration rate deserves discussion. Theoretically, the MAPK/ERK pathway activation, mitochondrial K_ATP_ channel opening, intracellular free radicals production, and lipid peroxidation contribute significantly to the cytoprotective mechanisms of IPC [23]. Therefore, the effects of co-treatment of IPC and antioxidants, such as Vitamin C [46,47], Vitamin E [48], and melatonin [49], have been investigated in animal myocardial I/R models. The results were inconclusive. Vitamin E pretreatment did not prevent myocardial I/R-induced lipid peroxidation and preserved the beneficial effect of IPC. Melatonin maintained the cardioprotective effect of IPC despite inhibiting lipid peroxidation. On the contrary to other antioxidants, vitamin C abolished the reduction in myocardial infarct size induced by IPC, and the proposed mechanism was that Vitamin C inhibited oxidative stress-induced MAPKs activation. In clinical settings, IPC effectively reduced ischemic chest discomfort during percutaneous coronary intervention [50]. CoQ10 supplementation promoted antioxidants in patients with coronary artery disease [51]. In consistent with the effects of Vitamin C, this is the first study showing that the combination therapy normalized the benefits of IPC and CoQ10. In the present study, the enhanced mitochondrial respiration at two hours after reperfusion in patients subjected to IPC was antagonized when IPC was applied in CoQ10-supplemented patients. This finding indicated that CoQ10 potentially prevents the protective effect of the IPC. It is possibly because CoQ10 is effective in preventing lipid peroxidation [15] and inhibiting the MAPK/ERK signaling pathway [52]. 

This study contains some limitations. Firstly, there are several methods of mitochondrial function assessment, including measurement of respiration rate and membrane potential [28]. In this study, the mitochondrial OCRs of PBMCs collected from systemic circulation were measured by the extracellular flux analyzer. Besides, instead of the systemic PBMCs, mitochondria can be obtained from the PBMCs of local circulation, such as a femoral vein or surgical drainage tube, and directly from the insulted skeletal muscles [10]. From the latter, assessment of mitochondrial alteration related to TQ-induced I/R is likely to be more prominent. Secondly, biochemical parameters indicating oxidative stress, inflammation, and apoptosis should be measured to specify the mechanisms of the two interventions. Thirdly, because of the variable bioavailability of CoQ10 used in supplementation, CoQ10 concentration in plasma or PBMCs should be assessed to determine the actual effectiveness of this CoQ10 formula and dosage. This finding would provide the direct relationship between circulating CoQ10 and its interaction with IPC in patients undergoing TKA. Next, changes in protein or gene expression of the lower extremity muscle during the I/R and postoperative muscle strength should be evaluated because the TQ-induced I/R injury significantly affects the localized skeletal muscle [10]. Lastly, this study contained a small number of subjects, possibly not adequate to detect differences of some outcomes with small effect size.

## 5. Conclusions

When interpreted in the context of a specific dose of CoQ10 and technique of IPC, IPC enhanced mitochondrial function by transiently increasing basal and ATP-linked respiration at two hours after reperfusion and CoQ10 provides analgesic effect by reducing postoperative pain at 24 h. Even though CoQ10 and IPC potentially prevent TQ-induced I/R injury and improve clinical outcomes, a combination of the two interventions possibly interferes with the beneficial effect of each intervention. Further trials investigating the potential mechanisms underlying the CoQ10 and IPC interaction and the mechanism of pain reduction following CoQ10 supplementation are suggested. 

## Figures and Tables

**Table 1:** Demographic data of knee osteoarthritis patients receiving placebo vs. CoQ10.

	Placebo (*n* = 24)	CoQ10 (*n* = 20)	Mean Difference (95% CI)
Female, *n* (%)	20 (83%)	16 (80%)	
Age (y), mean ± SD	66.5 ± 5.8	70.4 ± 6.3	3.9 (0.2, 7.6)
BMI (kg/m^2^), mean ± SD	26.0 ± 3.6	24.4 ± 3.9	−1.6 (−3.9, 0.7)
ASA, *n* (%)			
II/III	24 (100%)/0 (0%)	19 (95%)/1 (5%)	
Co-morbidity, *n* (%)			
Hypertension	15 (63%)	15 (75%)	
Diabetic mellites	3 (13%)	2 (10%)	
Dyslipidemia	11 (46%)	9 (45%)	
Current medication, *n* (%)			
β-blocker	1 (4%)	2 (10%)	
Metformin	2 (8%)	1 (5%)	
Statin	9 (38%)	8 (40%)	
Side of operation, *n* (%)			
Right/left	13 (54%)/11 (46%)	14 (70%)/6 (30%)	
Surgeon 1/surgeon 2	11 (46%)/13 (54%)	11 (55%)/9 (45%)	
Operation time (min), mean ± SD	115.5 ± 29.3	111.3 ± 22.9	−4.2 (−20.5, 12.0)
TQ duration (min), mean ± SD	87.6 ± 17.0	87.1 ± 15.9	−0.6 (−10.7, 9.5)
TQ pressure (mmHg), mean ± SD	252.9 ± 21.2	250.5 ± 13.2	−2.4 (−13.4, 8.6)
Intraoperative oxygen use, *n* (%)	7 (29%)	2 (10%)	
Intraoperative hypotension, *n* (%)	6 (25%)	4 (20%)	

## Data Availability

The authors confirm that the data supporting the findings of this study are available within the article.

## Abbreviations

ATP	adenosine triphosphate
ETC	electron transport chain
IPACK	interspace between the popliteal artery and capsule of the knee
IPC	ischemic preconditioning
I/R	ischemia and reperfusion
mPTP	mitochondrial permeability transition pores
NRS	numeric rating scale
OA	osteoarthritis
OCR	oxygen consumption rate
OXPHOS	oxidative phosphorylation
PBMC	peripheral blood mononuclear cell
ROS	reactive oxygen species
SBP	systolic blood pressure
TKA	total knee arthroplasty
TQ	tourniquet
